# Converging orifice used to control the discharge rate of spherical particles from a flat floor silo

**DOI:** 10.1038/s41598-023-27431-8

**Published:** 2023-01-12

**Authors:** Joanna Wiącek, Józef Horabik, Marek Molenda, Piotr Parafiniuk, Maciej Bańda, Mateusz Stasiak

**Affiliations:** Institute of Agrophysics, Polish Academy of Sciences, Doświadczalna 4, 20-290 Lublin, Poland

**Keywords:** Mechanical engineering, Computational science

## Abstract

The effect of the converging orifice geometry in a model silo on the discharge rate of monosized spherical particles was studied experimentally and numerically. The cylindrical container was equipped with interchangeable inserts with converging discharge orifices of various upper diameters in the upper base and a constant lower diameter in the lower base. Plastic PLA beads and agricultural granular materials: wheat, rapeseeds, and linseeds were tested. A series of discrete element method simulations corresponding to the performed experiments was conducted with a largely extended set of experimental discharge conditions. In the case of the constant thickness of the insert, the discharge rate initially increased with an increase in the half cone angle of the converging orifice and then the tendency reversed. In the majority of cases, the discharge rate through the converging orifice was higher than through the hopper with the same orifice diameter.

## Introduction

Questions of reliable flow of granular materials through horizontal orifices are the focus of interest in granular mechanics and technology. Despite the long standing investigations conducted by physicists and engineers, numerous effects remain obscure^1^. One of such effects is the influence of boundary conditions around the discharge gate on the flow pattern and the mass discharge rate (*MDR*) of granular material in a storage silo^2–4^. The *MDR* is one of the crucial parameters for the design and control of processes involving flow of granular materials and powders. A steady and precisely controlled flow rate is indispensable for preparing mixtures of materials in numerous branches. The boundary condition, i.e. the shape of the volume contained in the orifice and its vicinity, is a crucial factor determining the volume fraction and, consequently, the flow rate through the orifice^1,5,6^.

The flow rate through a horizontal orifice may be efficiently predicted by Beverloo’s equation^7^, which states that the mass discharge rate can be expressed as $MDR = C\rho_{b} \sqrt g (d - kd_{p} )^{5/2}$, where *d* is the orifice diameter, *d*_*p*_ is the particle diameter, *g* is the acceleration of gravity, *ρ*_*b*_ is the bulk density of discharging material, and *C* and *k* are the empirical discharge and shape coefficients, respectively. It has been revealed that the flow rate is different for small and large orifices (related to particle diameter), and the Beverloo relation breaks down for small orifices. Gella, Maza, & Zuriguel^8^ studied experimentally the effect of particle size on the mass flow rate from a model silo. The authors concluded that the relationship between the mass flow and the nature of contact interactions between particles, friction, or differences in kinetic energy per unit area is not trivial, and further research is necessary to clarify these questions. Beverloo, Leniger, & Van de Velde^7^ measured *MDR* during discharge of granular solids (mainly plant seeds) through an orifice in a flat bottom container. In such a configuration, stagnant material around the orifice forms a natural hopper where radial flow changes into a loose vertical stream of outflowing particles. A study on the effect of the cylindrical orifice geometry on the particle discharge rate was carried out for flat-bottomed silo by Zatloukal and Šklubalová^9^. Authors have confirmed a relationship between the discharge rate and the orifice size; however, they have also found a dependence of the flow rate on the orifice height. Zaki and Siraj^10^ have performed numerical simulations for three orifice shapes placed in the flat-bottomed cylindrical silo for spherical glass beads. Beverloo equation constants were calculated and the differences between the mass discharge rates for circular, triangular and square orifice were found. A high effect of the particle shape on the flow of particles discharged from the flat-bottomed silo has been reported by Hafez et al.^11^. Particle shape defines particle-to-particle interaction and relative mobility, which determine the discharge flow rate and the clogging behavior of granular solid.

In practical applications, silos with conical hoppers are frequently used, where no dead zone forms and discharging material slides along the smooth hopper surface. One of the first empirical equations predicting dependence of *MDR* on the conical hopper half cone angle α proposed by Rose & Tanaka^12^ is based on introduction into to the Beverloo equation the multiplicative factor comprising the impact of the half cone angle and the inclination of a stagnant zone boundary of the material inside the hopper. Saleh, Golshan, & Zarghami^4^ analyzed over twenty empirical models relating *MDR* to the hopper geometry. As concluded by the authors, no general rule has been established so far for the relationship between hopper half angle *α* and *MDR*.

Recently, reports have been published presenting numerical methods for designing hoppers with a varying contraction rate to maximize the mass discharge rate of granular material. The finite element method^13,14^ or the discrete element method^15^ with efficiency corroborated by experimental verification^16^ are used most often. Some results have shown that *MDR* can be increased by nearly 140% in a curved hopper, compared to a conical hopper with the same orifice size, hopper height, and silo diameter. Proper silo geometry may allow to control precisely the flow rate of granular material discharging the silo; however, understanding how to manipulate the mass discharge rate requires further research. That may have practical applications in metering, dosing or mixing.

Considering the results of above-mentioned studies, the objective of the reported project was to carry out a systematic study of the flow through a conical converging orifice with various values of thickness and half cone angle. A possibility of replacing the hopper bottom by the flat bottom equipped with converging discharge orifices in silo has been investigated. Motivation for the present study comes from the industrial flow of powders and grains in various devices. The converging parts, e.g. welding neck flanges, are common and important components of many practical apparatus used in the transport and processing of liquids and granular solids^17,18^.

So far no attempts have been made to use a numerical method for analyzing flow rate of granular materials through a conical converging orifice with various geometry. Therefore, series of the discrete element method simulations, supplemented by laboratory experiments have been performed. The specific appliance was designed for purpose of that project.

## Methods and materials

### Laboratory testing

**Figure 1:**
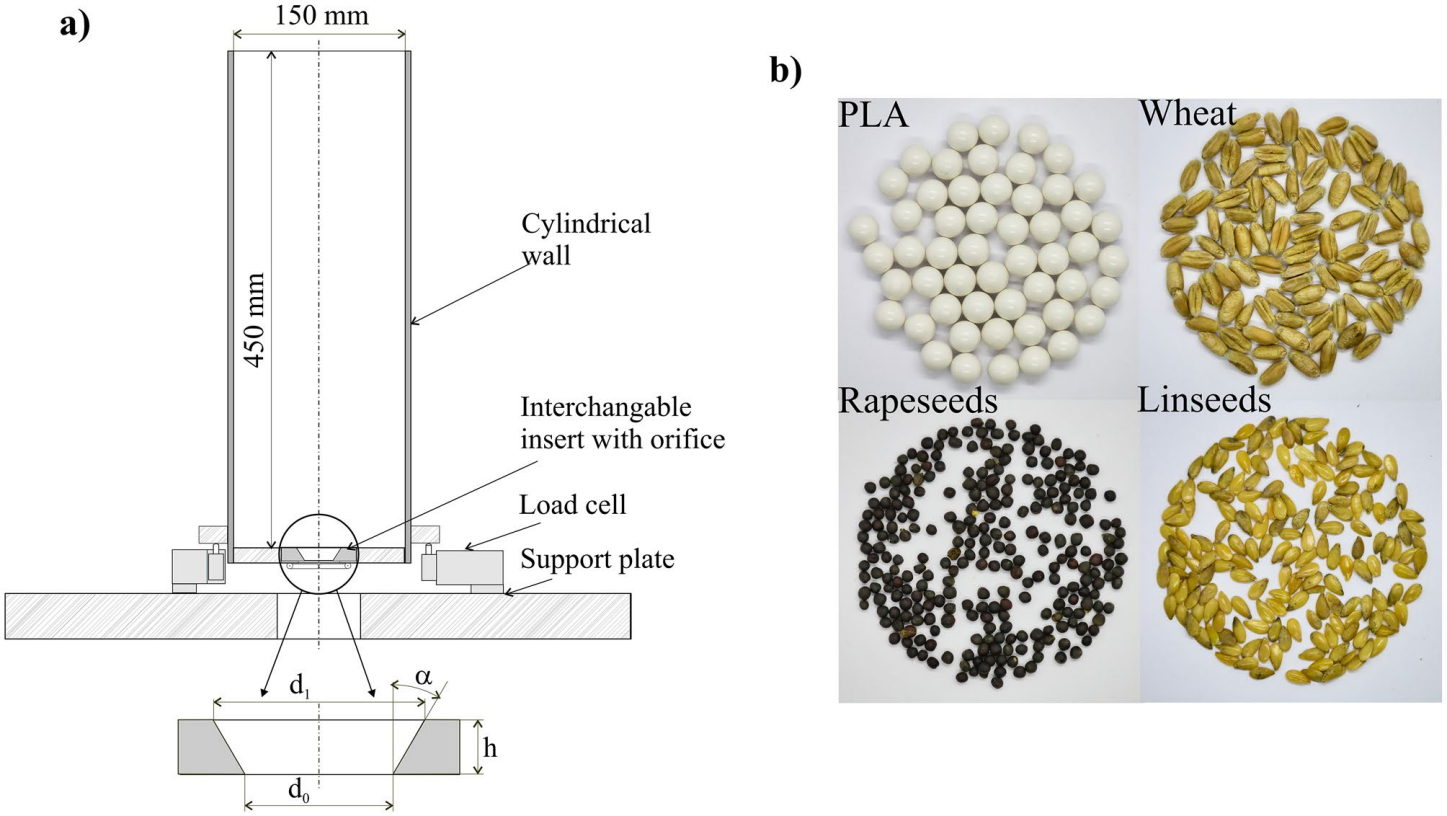
(**a**) Scheme of the model bin with an interchangeable inset housing the converging orifice used for testing the mass discharge rate, and (**b**) PLA particles and seeds used for the experiments.

The experimental silo has been used to measure the mass discharge rate *MDR*. The cylindrical flat bottomed container (Fig. 1a) was 150 mm in diameter and 450 mm high. The container wall was made of galvanized steel, while its flat floor was made of plywood. Plastic PLA beads with a diameter of 5.95 mm, *d*_*p*_, and the mass of 0.25 g were used as reference particles. The number of PLA particles in the sample was equal to 14,000. Wheat, rapeseeds, and linseeds were tested as agricultural granular particles (Fig. 1b, Table 1). The frictional parameters of particles were determined with use of the tilting table method (Table 2). The silo diameter was 25 times bigger than the largest particle diameter, which, according to the findings reported in literature, allowed neglecting the influence of the bin wall^19–21^. A repeatable filling procedure was adopted to maintain a similar geometrical bedding structure in subsequent tests. A sieve was placed axially on the top surface of the silo. The measured amount of particles was poured through the sieve. After completion of filling, the top free surface was leveled. The discharge gate was opened and the mass of particles leaving the container was measured until the discharge was completed. Indications of three load cells supporting the silo were used to determine change in the mass of silo and particles during discharge. The change in the mass of discharged particles was determined also from indication of one load cell supporting the receiving container (not included into Fig. 1a). The mean value of outputs of this two measuring methods was used to calculate the discharge rate. The container was equipped with interchangeable 3D printed plastic inserts of various thickness, *h*. The converging orifice in the flat bottom of the model silo was defined by three parameters: the lower diameter, *d*_0_, the upper diameter, *d*_1_, and the insert thickness, *h* (i.e. the distance between the lower and the upper edge of the orifice). The converging orifices with the lower diameter *d*_0_ of 32.5 mm and various upper diameters *d*_1_ were selected to verify the key finding of the DEM simulations. The insert was placed in the cylindrical hole of the flat floor made of plywood and aligned with the top surface of the bottom (Fig. 1a). The insert with the upper diameter of the orifice *d*_1_ = 32.5 mm, the lower diameter *d*_0_ = 52.5 mm, and the thickness *h* = 6 mm served as the reference flat orifice (α = − 60º). Three replicate experiments were performed for each material.

**Table 1:** Basic mechanical parameters of PLA particles and seeds used for experiments.

Material	Length (mm)	Thickness (mm)	Width (mm)	Bulk density *ρ*_*b*_ (kg m^–3^)	Mass of 1000 particles (g)
PLA	5.95 ± 0.01	5.95 ± 0.01	5.95 ± 0.01	1326.3 ± 8.1	250 ± 1.53
Wheat	6.44 ± 0.24	2.57 ± 0.18	2.99 ± 0.31	711.7 ± 5.7	40.5 ± 0.32
Rapeseeds	2.10 ± 0.19	1.86 ± 0.19	1.96 ± 0.20	681.3 ± 6.4	3.5 ± 0.03
Linseeds	4.96 ± 0.26	0.97 ± 0.05	2.41 ± 0.14	650.3 ± 1.5	6.2 ± 0.01

**Table 2:** Frictional parameters of PLA particles and seeds used for experiments.

Material	Particle–particle μ_*p-p*_	Particle-metal μ_*p-w*_	Particle-plastic insert μ_*p-b*_
PLA	0.47 ± 0.01	0.49 ± 0.01	0.21 ± 0.01
Wheat	0.15 ± 0.02	0.1 ± 0.01	0.1 ± 0.01
Rapeseeds	0.12 ± 0.02	0.13 ± 0.01	0.11 ± 0.01
Linseeds	0.22 ± 0.01	0.19 ± 0.01	0.15 ± 0.01

### DEM simulations

The DEM^22^ simulations have been performed with an assembly of 14,000 spherical particles with diameters randomly distributed in the range of 5.94–5.96 mm, with the mean, *d*_*p*_, of 5.95 mm, to reproduce the size of the PLA spherical particles applied in the experiments as the reference material. The numerical geometry mimicked the experimental setup. The thicknesses *h* of the inserts were tested in a range from 0 to 100 mm. The majority of them were normal multiplicities of the particle mean diameter. The lower diameter *d*_0_ ranged from 19 to 55 mm and the upper diameter *d*_1_ ranged from 32.5 to 72 mm, providing the half cone angle ranging between 4 and 90º. The reference lower diameter *d*_0_ of the orifice was 32.5 mm. The flat orifice with *d*_1_ = 32.5 mm (*d*_0_ > *d*_1_) served as a reference orifice providing a non-disturbed discharge. The discharge through conical hoppers with the same half cone angle as that of the converging orifice provided additional reference data of the mass discharge rate. The orifice diameter of the hopper was 32.5 mm and the upper diameter was 150 mm.

The Hertz-Mindlin no-slip contact model was applied for simulations following the Hertz theory^23^ as the default model used in the EDEM software package^24^. The material parameters of the particles were taken to reproduce the properties of the PLA particles: solid density *ρ*  = 2212 kg/m^3^, Young’s modulus *E* = 8.8 GPa, and Poisson’s ratio ν = 0.25^25^. The frictional parameters between particles μ_*p-p*_ = 0.47, between particle and wall μ_*p-w*_ = 0.49, and between particle and bottom (plastic insert) μ_*p-b*_ = 0.21, as well as the coefficient of restitution *e* = 0.3 were determined experimentally. A default value of the rolling friction of 0.01 of EDEM software was applied for simulations. The walls of the silo were modelled with density *ρ*  = 7800 kg/m^3^, Young’s modulus *E* = 200 GPa, and Poisson’s ratio ν = 0.25, which were material parameters of the steel.

**Figure 2:**
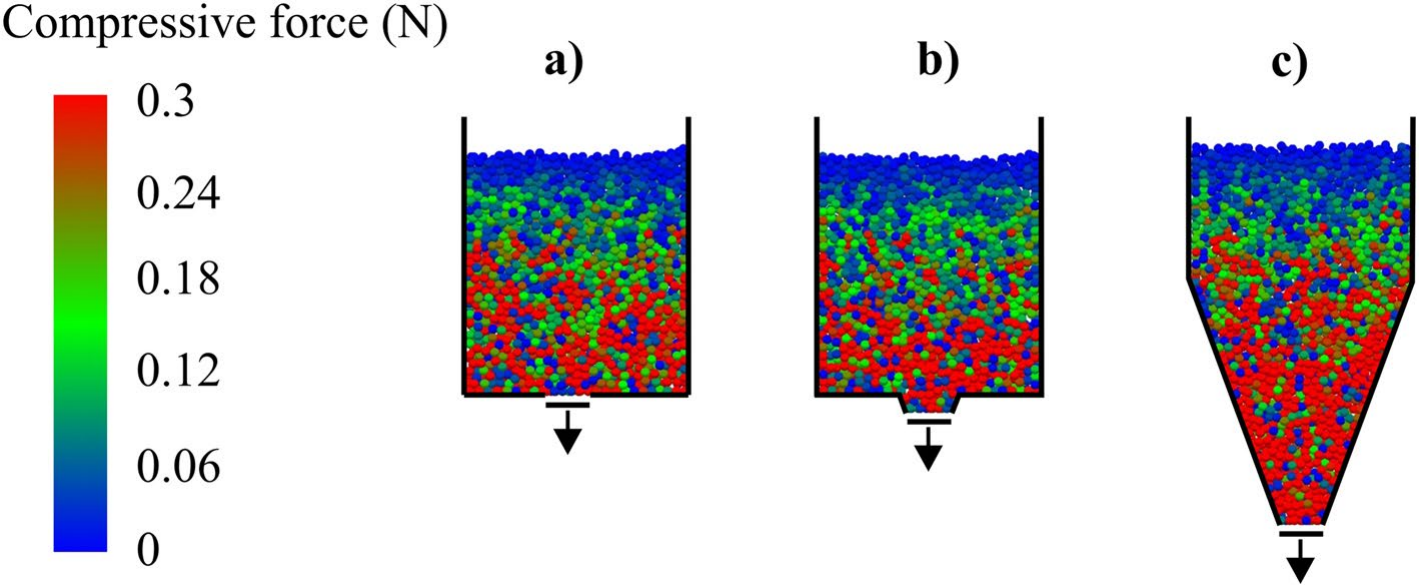
Visualization of the contact forces between particles in the middle slice of model silo at rest: (**a**) flat orifice, (**b**) converging orifice, (**c**) hopper with the same *α* as that of the converging orifice.

Particles were generated inside the model silo. The particles were then discharged through a centrally located flat orifice, a converging orifice, or a conical hopper (Fig. 2). The simulations were performed with a time step of 1.6∙10^–6^ s with use of EDEM software package^24^.

The simulations were performed according to the following scheme of setting the converging orifice parameters:

- (1) *d*_1_ = var., *α*  = var., *d*_0_ = const., *h* = const.,
- (2) *d*_0_ = var., *d*_1_ = *d*_0_ + const., *h* = const., *α*  = const.,
- (3) *d*_0_ = var., *α* = var., *d*_1_ = const., *h* = const.

## Results

### Discharge scheme No. (1) d_1_ = var., α= var., d_0_ = const., h = const.

**Figure 3:**
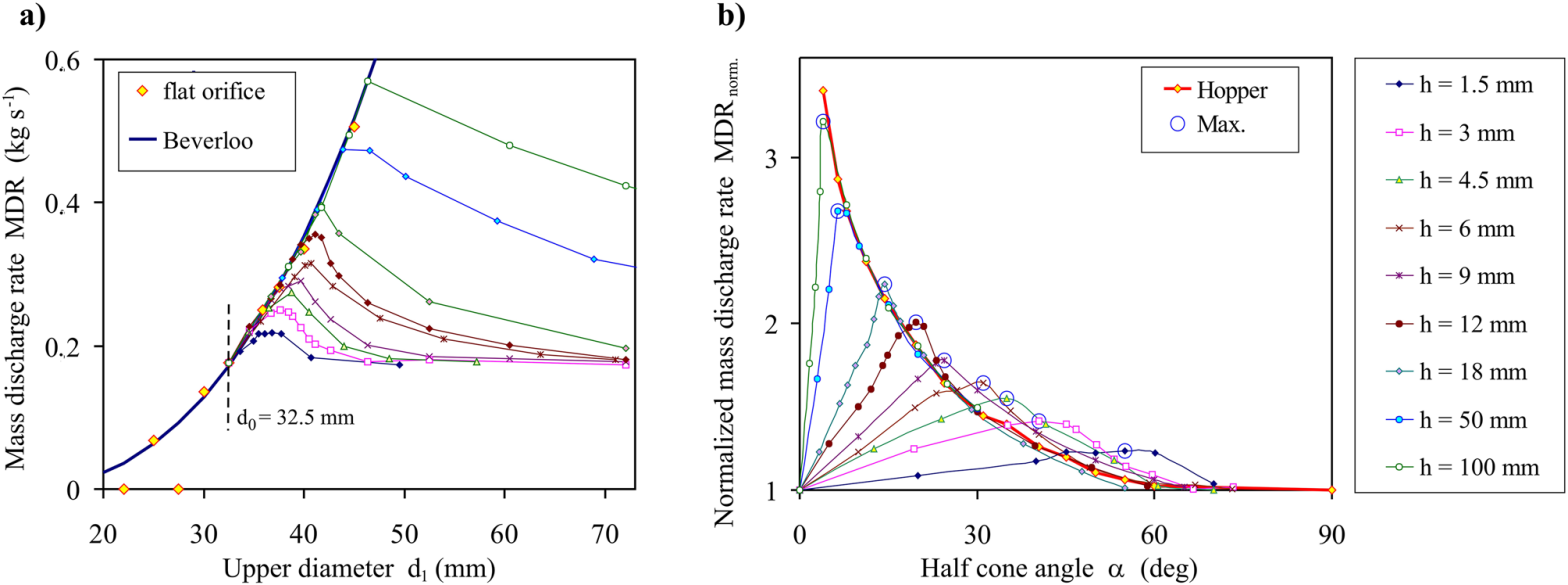
(**a**) Mass discharge rate *MDR* influenced by the upper diameter of the orifice *d*_1_ for *d*_0_ = 32.5 mm and several values of the orifice thickness *h*. Discharge through the flat orifice approximated by Beverloo’s equation for *C* = 0.54 and *k* = 1.65, and (**b**) simulated *MDR*_*norm.*_ normalized by the mass discharge rate through the flat orifice with *d*_1_ = *d*_0_ = 32.5 mm as a function of the half cone angle *α*.

The preliminary DEM simulations performed for the flat orifice (*d*_0_ > *d*_1_) with the diameter *d*_1_ in the range from 19 to 35 mm indicated that the threshold orifice size providing an undisturbed flow of material from the silo was 32.5 mm. Therefore, in the further study, the lower diameter *d*_0_ = 32.5 mm was applied for the simulations. The DEM simulated relationship between the mass discharge rate *MDR* and the upper diameter of the converging orifice *d*_1_ for *d*_0_ = 32.5 mm and several values of the insert thickness *h* are shown in Fig. 3a. The *MDR* calculated according to Beverloo’s equation with parameters *C* = 0.319 and *k* = 1.65 applied for the flat orifice was appended for comparison. For all thicknesses of the insert, the values of *MDR* initially followed Beverloo’s approximation until the maximum *MDR* was reached. The maxima of *MDR* and corresponding *d*_1_ increased with the increase in the insert thickness. They were located close to Beverloo’s approximation. Next, after surpassing the maximum, the *MDR* decreased initially rather fast and with growing *d*_1_ tending to a horizontal asymptote. The asymptotic value of the *MDR* for sufficiently high *d*_1_ (i.e. for *α* tending to 90º) is the *MDR* for the flat orifice of *d*_1_ = 32.5 mm.

Figure 3b shows a change in the normalized mass discharge rate (*MDR*_*norm.*_) with the increasing half cone angle *α* of the converging orifice. Mass discharge rates were normalized by the mass discharge rate determined for the flat orifice of *d*_1_ = 32.5 mm. For all tested thicknesses, the *MDR*_*norm.*_ initially increased with the increasing *α*. After the maximum was reached at *α*_*crit.*_, the mass flow rate monotonically declined to the *MDR* obtained for the flat reference orifice (i.e. *MDR*_*norm.*_ → 1). The highest maximum of the *MDR*_*norm.*_ (> 3) was obtained for *α*_*crit.*_ = 4º and *h* = 100 mm. The maximal values of *MDR*_*norm.*_ decreased with the decrease in the thickness of the insert and were noted for the higher half cone angle *α*_*crit*_. For small values of *α*_*crit.*_ the maxima *MDR*_*norm.*_ obtained for the converging orifice were 5% lower than those obtained for the hopper with the same half cone angle *α* and the same orifice diameter of 32.5 mm, while the maxima for *α* > 20º were approximately 10% higher than those for the hopper.

The course of the relationships *MDR*_*norm.*_(*α*) may be interpreted in the light of the Jenike criterion for the flow pattern in a conical hopper as dependent on the angle of internal friction and on the *α* value^14,24^. In the case of a steep hopper (low *α*), a mass flow takes place. After an increase in *α* to a limiting value, the flow pattern changes into a funnel flow. A further increase in *α* leads to formation of a stable dead zone with a converging flow identical to that present in a flat floor silo.

**Figure 4:**
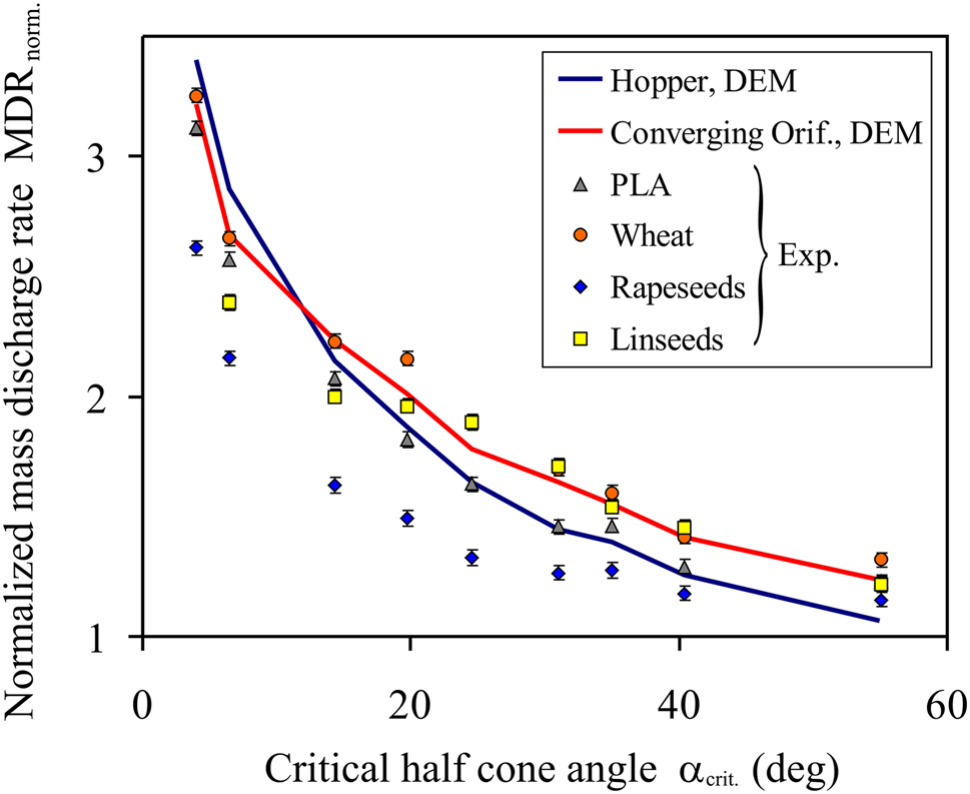
Comparison of the experimental and DEM simulated values of the normalized mass discharge rate *MDR*_*norm.*_ obtained for different alues of the critical half cone angle *α*_*crit.*_.

The results of the laboratory tests performed for four granular materials discharged from the converging orifice with geometry providing the maximum *MDR* in the DEM simulations were compared with the numerical results obtained for the same geometry of the converging orifice and for the hopper (Fig. 4). The experimental and numerical results were in reasonable agreement. Both of them showed the same tendency of a decrease in *MDR*_*norm.*_ with the *α*_*crit.*_ increase. Most of the experimental results were located very close to the results of the simulations performed for the converging orifice. The values of *MDR*_*norm.*_ for rapeseeds were lower than those for the other materials, which should be attributed to the over twice larger difference in the size of the seeds. This is consistent with the findings reported by Gella, Maza, & Zuriguel^8^, who indicated different profiles of the solid fraction in the vicinity of the orifice in assemblies of spheres of the same material and a four-fold difference in the diameter. The fairly close courses of the *MDR*_*norm.*_(*α*_*crit.*_) relationships for the hopper and for the converging orifice indicate a crucial role of the geometry of near proximity of the outlet for the flow rate of discharging material. Conditions on the hopper wall further from the outlet seem to have only a weak influence on the flow rate.

**Figure 5:**
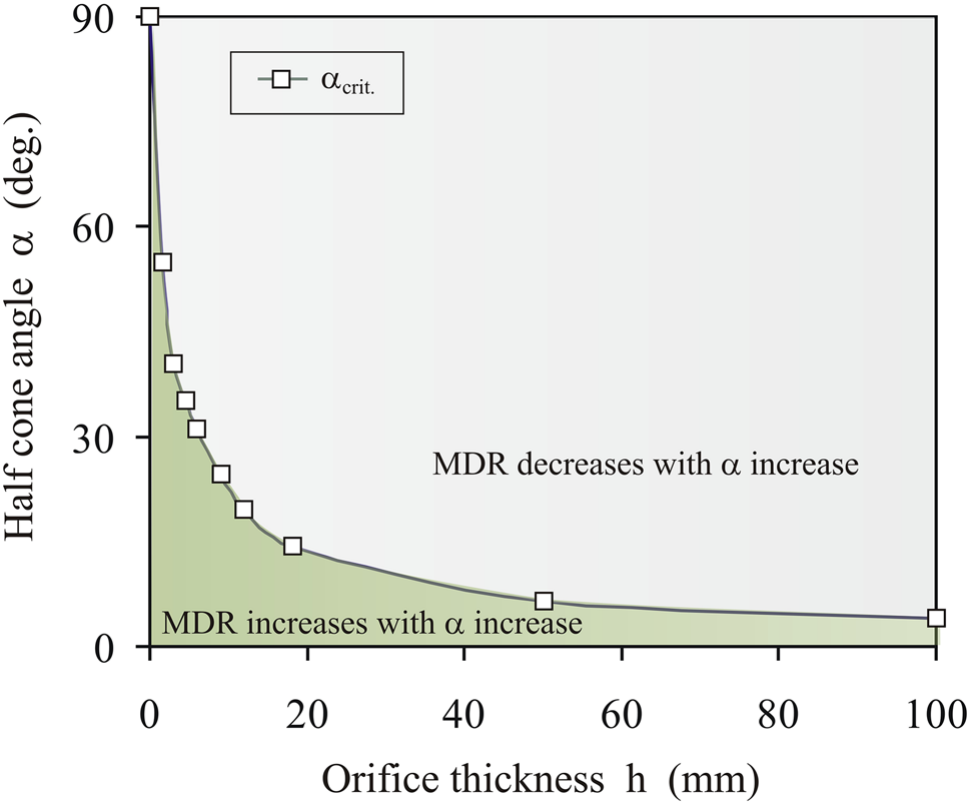
Relationship *α*_*crit.*_(*h*) separating the geometry of the converging orifice (*h,α*) into two regions of increase (*α* < *α*_*crit.*_) and decrease (*α* > *α*_*crit.*_) of the mass discharge rate *MDR* with *α* increase determined for *d*_0_ = 32.5 mm.

The analysis of the dependence of the *MDR* on *α* determined for *d*_0_ = 32.5 mm and for all the tested values of the parameters *d*_1_ and *h* has shown that the dependence of the critical value of the half cone angle on the orifice thickness *α*_*crit.*_(*h*) separated the geometry of the converging orifice (*h,α*) into two regions with respect of the dependence of the *MDR* on *α* for *d*_0_ = const.: (1) the *MDR* increasing with *α* increase for *α* ≤ *α*_*crit.*_ and (2) the *MDR* decreasing with *α* increase for *α* > *α*_*crit.*_ (Fig. 5).

### Discharge scheme No. (2) d_0_ = var., d_1_ = d_0_ + const., h = const., α = const.

**Figure 6:**
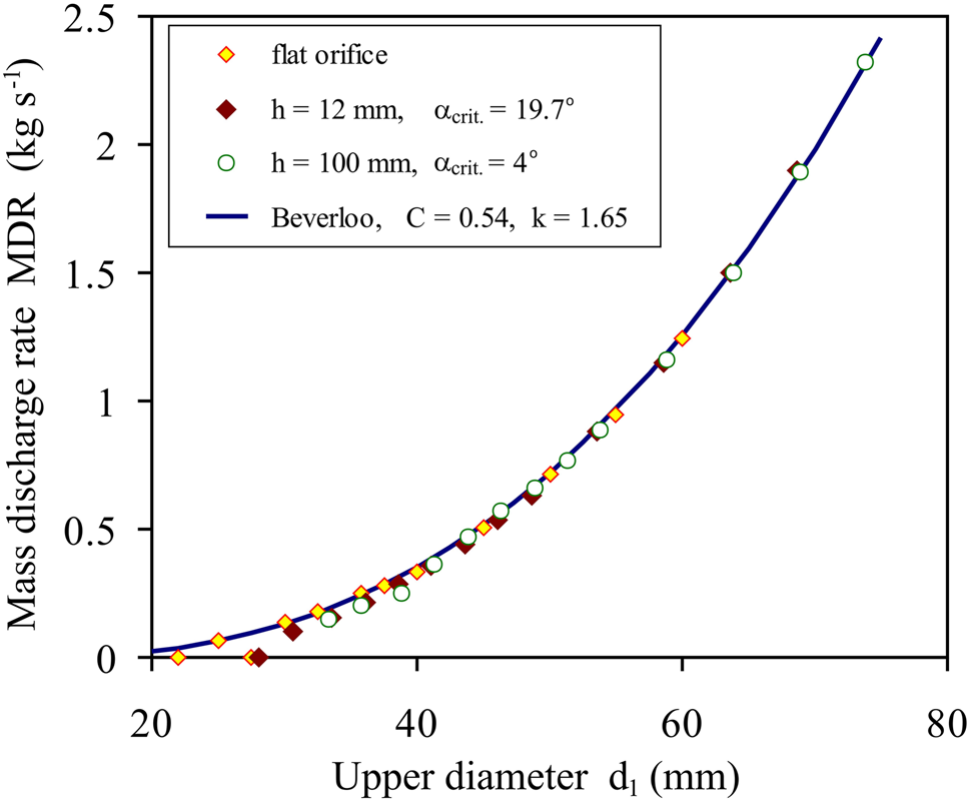
Comparison of simulated *MDR*(*d*_1_) relationships performed for the flat orifice and two values of the orifice thickness *h* of the converging orifice and the half cone angle *α*_*crit.*_ providing the maximum discharge rate with predictions of Beverloo’s equation.

Figure 6 presents the mass discharge rate *MDR* as the function of the upper diameter *d*_1_ of the converging orifice for two values of the insert thickness *h* and the half cone angle *α*_*crit.*_ providing the maximum discharge rate, compared with the results obtained for the flat orifice and Beverloo’s relationship. The critical value of the half cone angle *α*_*crit.*_ depended only on the insert thickness *h*. Contrary to the relationships shown by simulation scheme No. 1 (*d*_0_ = 32.5 mm, *d*_1_ = var.) (Fig. 4), the relationships obtained using scheme No. 2 (*d*_1_ = var., *d*_1_ = *d*_0_ + const., *h* = const., *α* = const.) followed Beverloo’s relationship very well. This means that the relationship *MDR*(*d*_1_) obtained for the converging orifice with *α* = const. ≤ *α*_*crit.*_ followed Beverloo’s relationship obtained for the flat orifice.

### Discharge scheme No. (3) d_0_ = var., *α* = var., d_1_ = const., h = const.

**Figure 7:**
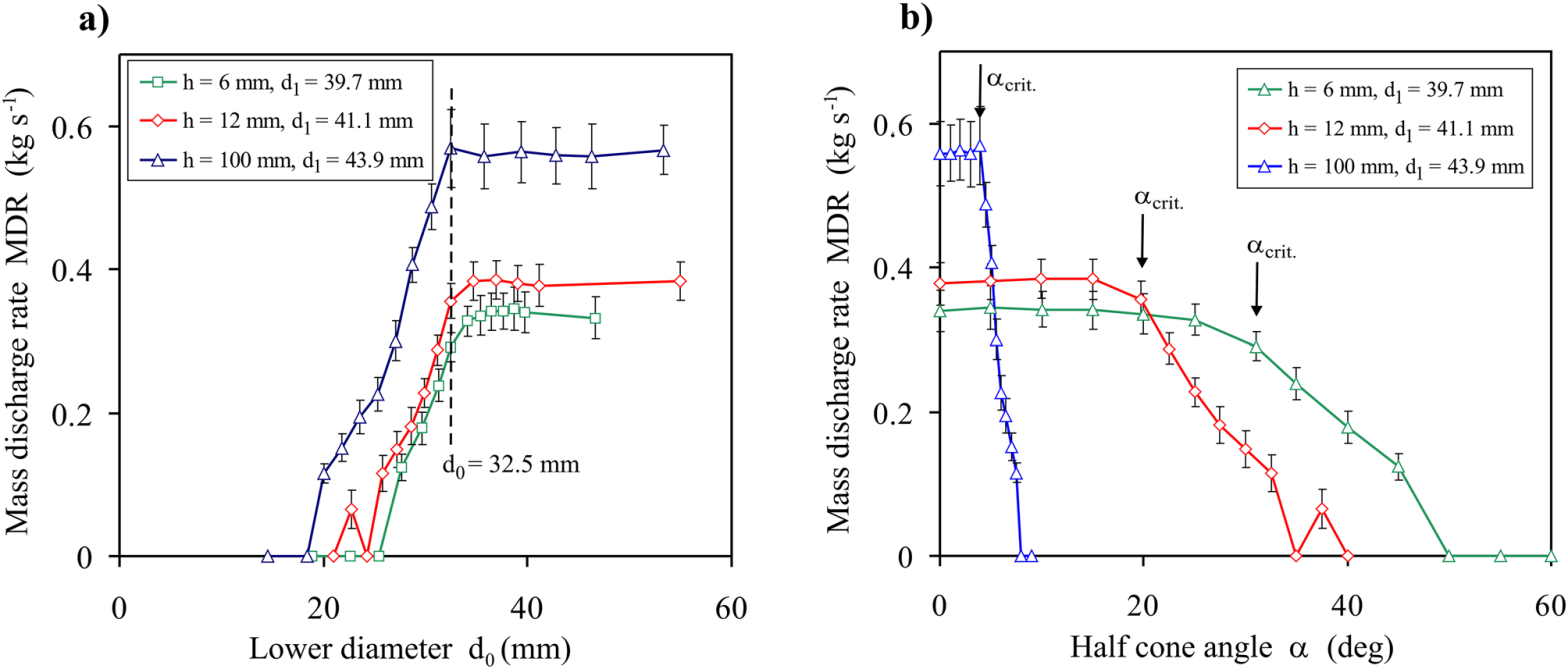
Mass discharge rate *MDR* vs.: (**a**) the lower diameter of the orifice *d*_0_, (**b**) the half cone angle of the orifice *α* for three different values of the thickness *h* of the converging orifice and the upper diameter *d*_1_ providing maximum *MDR* for *d*_0_ = 32.5 mm.

The simulations performed according to the third scheme of setting the orifice parameters illustrate clearly the limits of the influence of the upper and the lower diameter of the converging orifice on the *MDR*. Figure 7a presents the *MDR*(*d*_0_) relationship and Fig. 7b shows the *MDR*(*α*) relationship, averaged over ten moments of time, for three different values of the thickness *h* and the upper diameter *d*_1_. In the case of *h* = 100 mm, the clear maximum *MDR* was observed for *d*_0_ = 32.5 mm followed by the plateau for *d*_0_ > 32.5 mm. For *h* = 12 and 6 mm, the dependence was more diffused and the plateau started at *d*_0_ a bit larger then 32.5 mm. For *d*_1_ = const., the *MDR* increased with *d*_0_ up to its maximum/plateau and remained almost constant with the further increase in *d*_0_ (Fig. 7a). Substituting the *d*_0_ variable with the corresponding half cone angle *α* under the condition *d*_1_ = const., it can be observed that the *MDR* remained almost constant for *α* ≤ *α*_*crit.*_ and decreased with the *α* increase for *α* > *α*_*crit.*_ (Fig. 7b). Scatter of the *MDR* illustrated in Fig. 7 as the standard deviation bars disturbed precise determination of *α* initiating the plateau. The difference in the course of dependencies presented in Figs. 3 and 7 results from applying the different independent *x* variable: *d*_1_ in Fig. 3a and d_0_ Fig. 7a. Additionally, the half cone angle *α* applied in Fig. 3b and Fig. 7b depends in different way on the variables *d*_0_ and *d*_1_ ($\alpha = \tan^{ - 1} {{((d_{1} - d_{0} )} \mathord{\left/ {\vphantom {{((d_{1} - d_{0} )} {2h}}} \right. \kern-0pt} {2h}})$). The *MDR*(*α*(*d*_0_)) relationship can be converted into the *MDR*(*α*(*d*_1_)) relationship applying superposition of relationships obtained according to the Discharge schemes No. 3 and No. 2.

### Dense and loose flow through the orifice

**Figure 8:**
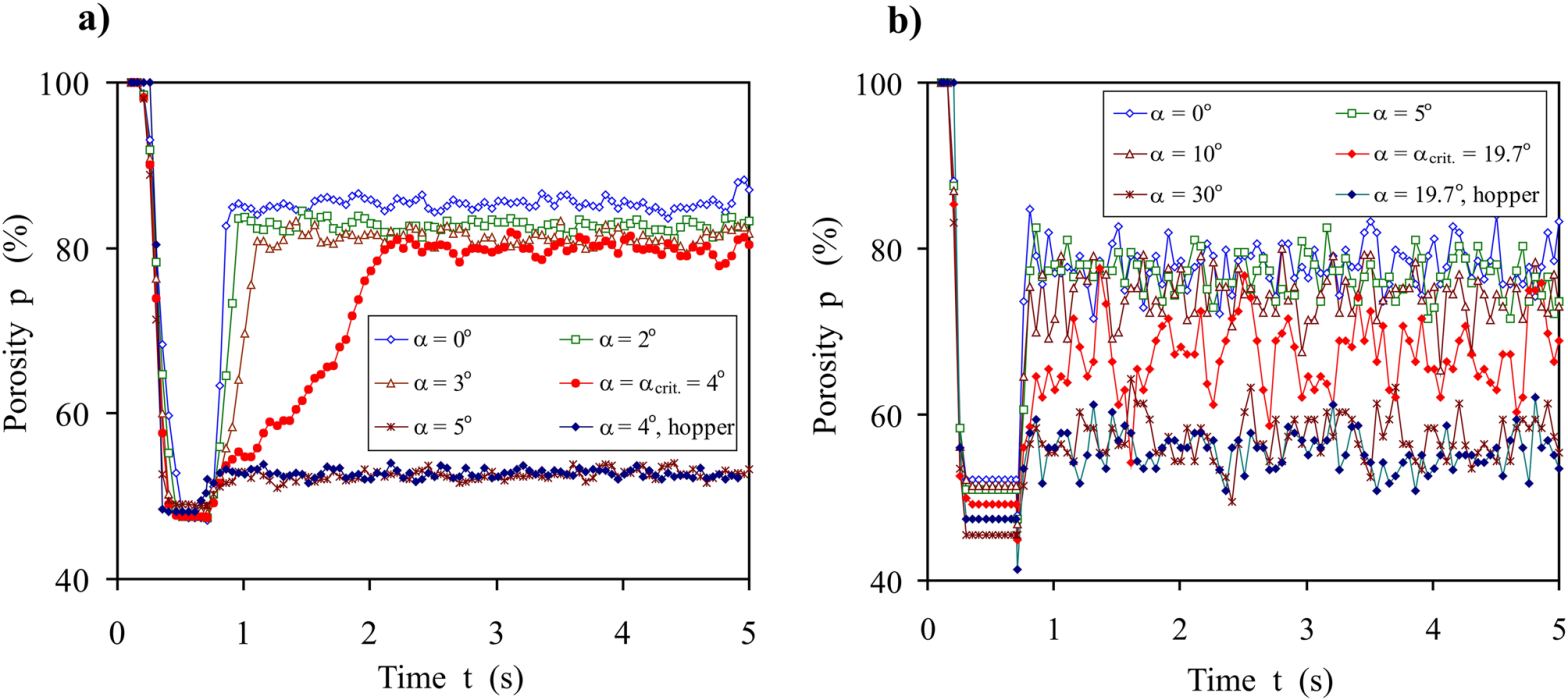
Porosity of the granular assembly inside the volume of the orifice of *d*_0_ = 32.5 mm vs. time during filling, detention, and discharge for: (**a**) *h* = 100 mm, (**b**) *h* = 12 mm.

Figure 8 shows changes in the mean porosity of the assembly of spherical particles determined in the volume of the orifice of *d*_0_ = 32.5 mm, for insert thickness of 100 mm (Fig. 8a), and 12 mm (Fig. 8b), at detention, after filling, and during commencement of the discharge. Porosity is defined as the ratio of the volume of pores to the volume of the assembly. The time variation of porosity in the volume of the orifice for several values of *α* has been shown. After filling, the porosity was approximately 48% in static conditions. For the insert with *h* = 100 mm, the discharge commencement produced a sharp increase in the porosity to a value dependent on *α* (Fig. 8a). For *α* values below 4°, the increase was nearly immediate. A further increase in *α* to 4° produced a substantial change in the *p*(*t*) relationship with a switch in porosity lasting for approximately 1.4 s. The porosity of the material flowing through the volume of the converging orifice was approximately 83% for *α* ≤ 4° and 53% for *α* ≥ 5°. The seemingly slight increase in *α* from 3° to 4° and subsequently to 5° produced substantial changes in the behavior of the material. The limiting value of the half cone angle was *α* = * α*_*crit*._ = 4°. The porosity inside the corresponding volume of the hopper of the half cone angle *α*  = 4° during the discharge was 53%, i.e. it was the same as the values of a dense flow obtained for the converging orifice with *α* > *α*_*crit.*_. The same tendency for changes in the porosity was observed for the insert with *h* = 12 mm and *α*_*crit.*_ = 19.7º (Fig. 8b). In this case, the relationships were not as clear as for *h* = 100 mm due to relatively big scatter of data resulting from discrete nature of the process averaged over eight times lover volume.

**Figure 9:**
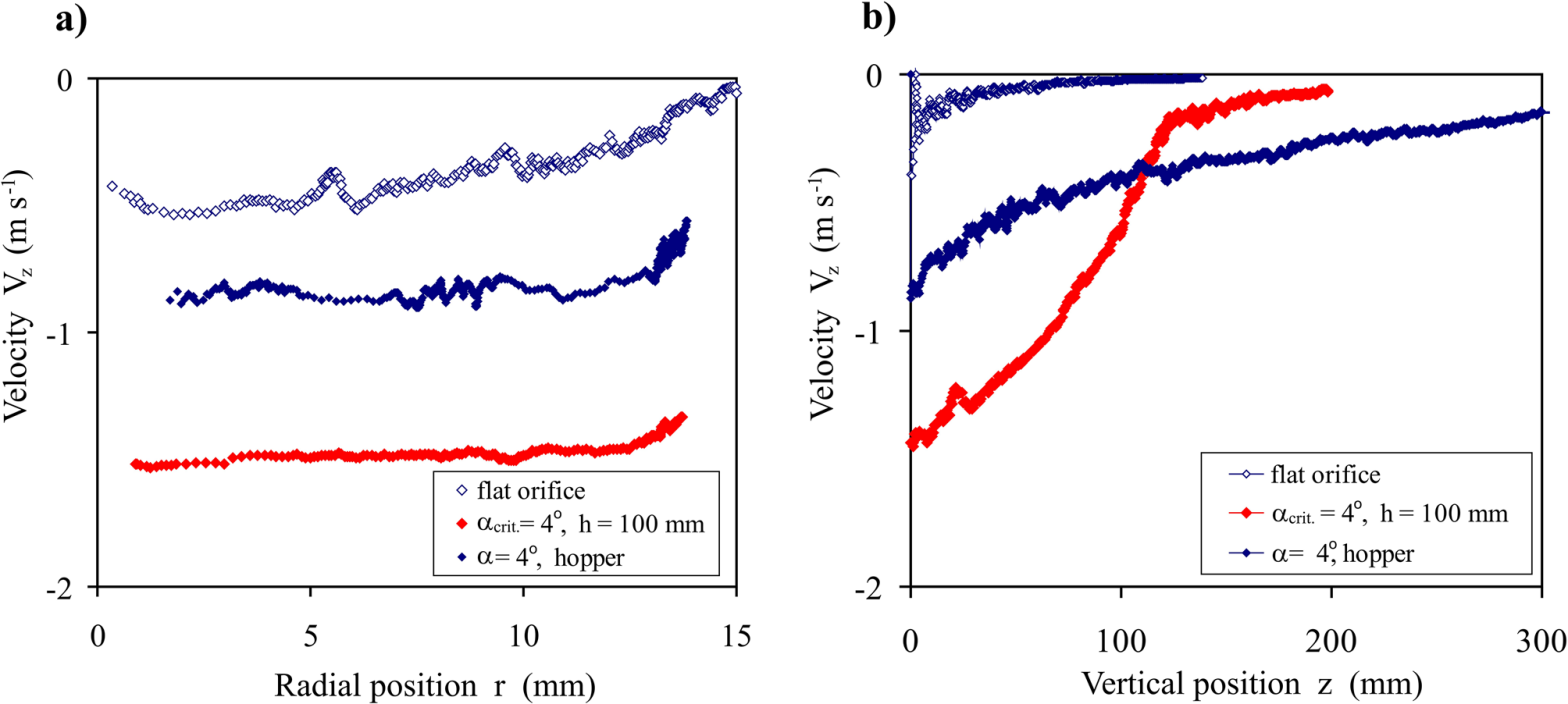
Profiles of the vertical velocity *V*_z_ averaged over ten moments of time, for the orifice of *d*_0_ = 32.5 mm: (**a**) in radial direction *r* at the level of the bottom edge of the orifice, (**b**) in vertical direction *z* (averaged over the cross-section of the orifice).

The comparison of the profiles of velocity *V*_*z*_ of particles during discharge for the flat orifice, converging orifice, and hopper with the same *α* and *d*_0_ (Fig. 9) explains the cause of the increase in the mass discharge rate through the converging orifice to values obtained for the hopper. For the converging orifice, at the level of the bottom edge of the orifice the particle velocity was approximately twice higher than the velocity of particles leaving the orifice (Fig. 9a). Figure 8a shows that the porosity in the converging orifice was also approximately twice higher than in the hopper. Therefore, the mass discharge rate, the product of the particle velocity and the bulk density, was similar for the converging orifice and the hopper with the same half cone angle *α*.

Profiles of the particles velocity *V*_z_ in the vertical direction have shown that the highest particles acceleration occurred in the converging orifice (Fig. 9b). Increase in the porosity during commencement of discharge through the converging orifice softened the structure of the bulk of particles, and, consequently, made accelerating particles easier due to gravity. Finally, it resulted in higher velocity at the level of the bottom edge of the orifice. Softening of the structure of the bulk of particles in the volume of the converging orifice with a thickness of a few particle diameters ensures the same mass discharge rate as the discharge of the dense structure of the bulk of particles through the hopper. This means that, by applying different geometries of the orifice, a similar mass discharge rate can be achieved by means of a stream of more densely packed particles with a lower particle velocity or a stream of more loosely packed particles with a higher particle velocity.

## Discussion

The need of deeper understanding of the kinematic transition region near the outlet in the silo is important for a precisely controlled discharge rate^1,5^. Therefore, the orifice dimensions were selected as variables to study discharge through the converging orifice.

The converging orifice can be considered as an extremely simplified curved hopper reduced into two segments: a flat floor and a short part of the hopper. Studies on the effect of the geometry of a conical converging orifice on the mass flow rate of granular material is scarce. Therefore, in this project, the results obtained for silos with conical hoppers were considered as a reference point. In the majority of cases, the flow rate through the converging orifice is higher than through the hopper with the same orifice diameter. Hence, the conical hopper may be replaced by flat bottom equipped with converging orifice with a smaller diameter to obtain the same discharge rate. The values of the *MDR* obtained for the converging orifice were located close to these provided by the hopper and considerably lower than the values provided by curved hopper, presented by Huang et al.^16,26^ and Guo et al.^14^.

The main novelty of the study is the indication of the hyperbolic type relationship between the half cone angle *α*_*crit.*_ and the thickness of the insert with converging orifice *h* separating the geometry of the converging orifice into two regions with respect of the dependence of the *MDR* on *α* for *d*_0_ = const.: (1) the *MDR* increasing with *α* increase for *α* ≤ * α*_*crit.*_ and (2) the *MDR* decreasing with *α* increase for *α* > *α*_*crit.*_. The results of this study corroborated the observation that the flow mode (bulk density of the stream and particle velocity) of granular material through a conical converging orifice depends on the half cone angle of the orifice. For *α* < *α*_*crit.*_, the discharge commencement produces a rapid increase in the porosity of the material in the volume of the orifice associated with the higher particle velocity. Attaining *α* =* α*_*crit.*_ produced a substantial change. The increase in porosity with the discharge time was much slower and nearly linear. Slight surpassing *α*_*crit.*_ (by one or two degrees) allowed a denser flow with a lower particle velocity.

At the flat floor of the bin, a dead zone is formed generating a natural hopper. In this area, the flow direction changes from vertical to converging, which is associated with softening of structure of the material. In a hopper, the change in the direction of particle movement is much smoother, which results in much lower dilation and acceleration along the straight line of particle movement. Despite such a big difference in the characteristics of particle movement between the converging orifice and the hopper, the mass discharge rate may be similar for the same half cone angle and appropriately adjusted height of the converging orifice. As concluded by Gella, Maza, & Zuriguel^8^, it is difficult to definitively state which specific property of particles is responsible for the macroscopic changes observed in the system. The relationship among all these magnitudes is not trivial, and further research is necessary to clarify these questions. Understanding how to manipulate and control the mass discharge rate may have a positive impact on the productivity and quality of industrial unit operations.

## Conclusions

The following detailed conclusions were drawn:

1. Material discharges in the dense (*α* > *α*_*crit.*_, porosity ≈ 60%) or loose (*α* ≤ *α*_*crit.*_, porosity ≈ 80%) flow mode depending on the insert thickness *h* and the angle of inclination of the generatrix of the converging orifice *α*. The maximal normalized mass discharge rate *MDR*_*norm.*_ decreased from 3.2 for *h* = 100 mm and *α* = 4º to 1.2 for *h* = 1.5 and *α* = 55º. In the majority of cases, the flow rate through the converging orifice is higher than through the hopper with the same orifice diameter.
2. For *d*_0_ = const. the critical value of the half cone angle *α*_*crit.*_ depended only on the insert thickness *h*. For *α* ≤ *α*_*crit.*_ the mass discharge rate followed Beverloo’s relationship obtained for the flat orifice. The hyperbolic type dependence of the critical value of the half cone angle *α*_*crit.*_ on the insert thickness separated the geometry of the converging orifice (*h,α*) into two regions of opposite reaction of the mass discharge rate *MDR* to *α* increase: (1) increase of the *MDR* with *α* increase for *α* < *α*_*crit.*_ and, (2) decrease of the *MDR* with *α* increase for *α *> * α*_*crit.*_.
3. The tendencies observed for the monodisperse assembly of spherical particles were preserved when beddings of wheat, lineseeds, and rapeseeds were tested. However, closer convergence of the results of the experiments and simulations would require fine tuning of the simulation parameters. The geometrical and mechanical parameters of real particles are far from those of a perfect sphere, which results in this discrepancy.
4. The results of the reported study show that the application of proper orifice geometry may allow precise control of the flow rate of granular material discharged from the silo. The fairly close compliance between the results of the experimental measurements and the simulations shows that DEM can be used to design equipment in systems involving granular flow.

## Data Availability

All data generated or analyzed during this study are included in this published article. Further detailed information on the datasets elaborated during the current study is available from the corresponding author and can be provided on reasonable request.
